# Total penile glans amputation following circumcision: A case report of a dramatic complication

**DOI:** 10.1016/j.eucr.2021.101905

**Published:** 2021-10-18

**Authors:** Ousmane Sow, Cyrille Ze Ondo, Alioune Sarr, Babacar Sine, Cheikh Becaye Gassama, Alain Khassim Ndoye

**Affiliations:** Urology-Andrology Department, Aristide Le Dantec Hospital, Dakar, BP: 3001, Senegal

**Keywords:** Glans amputation, Penis, Circumcision, Meatoplasty

## Abstract

Circumcision is one of the most commonly performed surgical procedures. As traditional ritual circumcisions are still practiced in the community, in Senegal the majority of circumcisions are performed in hospitals. We report the case of a 9-year-old boy who underwent a total amputation of the glans after a circumcision in a pharmacy by an unqualified agent. A meatoplasty was performed and the postoperative course were uneventful.

## 1 Introduction

Circumcision is the commonest operation performed on young boys**^1^****.** There are multiple complications that can occur following circumcision, ranging from the insignificant to the tragic**^1^****.** When performed by an experienced operator, circumcision is usually a safe and simple operation. In Senegal, circumcision is regarded as a customary ritual and is mostly done by nurses, the traditional circumcisers with medical doctors being involved less often. As a result of this, many complications arise ranging from minor injuries which are usually not reported immediately to catastrophic penile amputations. We report a case of total amputation of the glans occurring during circumcision, which was treated with meatoplasty.

## 2 Case presentation

**Fig. 1:**
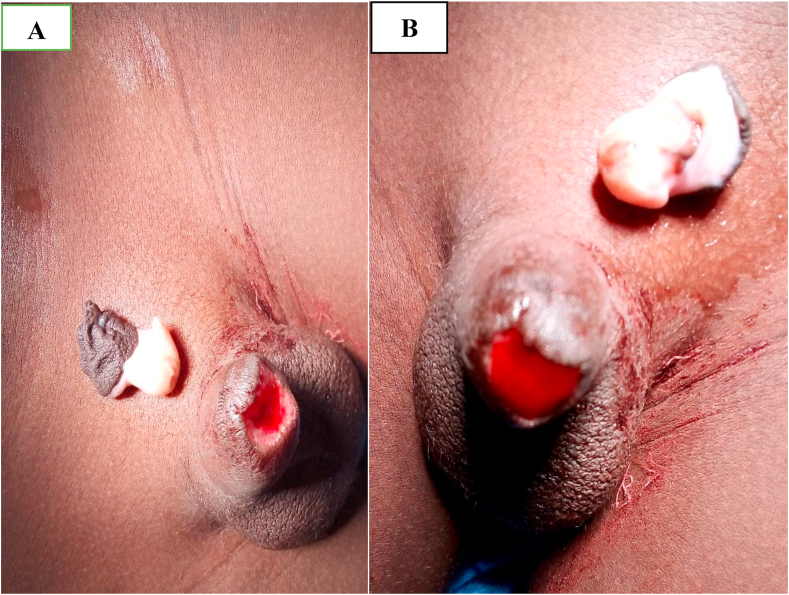
**(A)** Complete section of the glans and prepuce at the balanopreputial sulcus (**B)** The section slice showed bleeding.

**Fig. 2:**
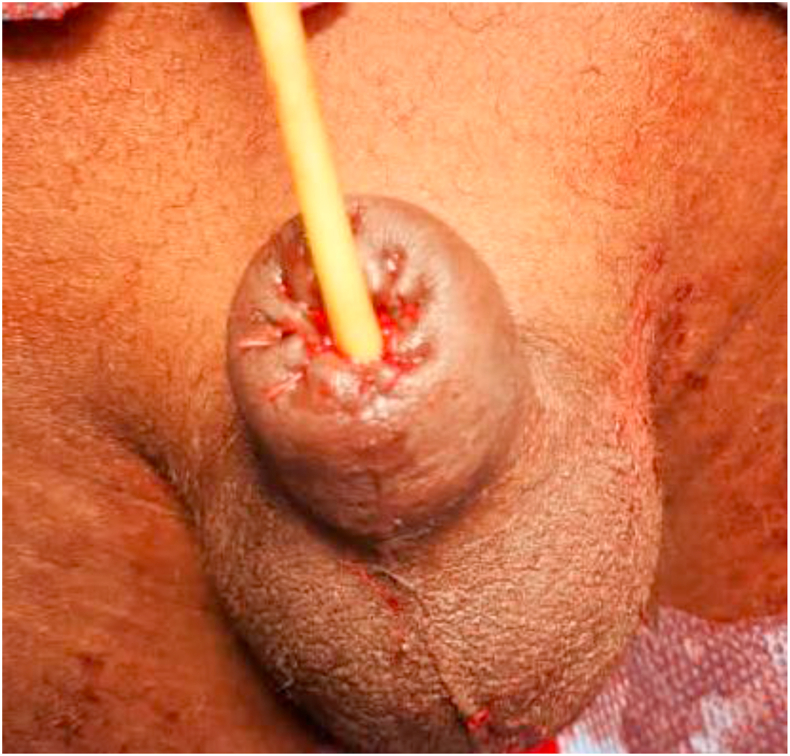
Final aspect of a meatoplasty.

**Fig. 3:**
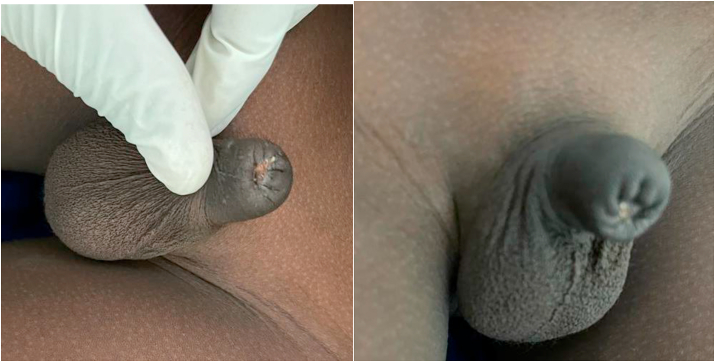
After a 2-month follow-up, glans penis and coronal sulcus absent with a pinhole urethral meatus with scaring at the distal end.

A 9-year-old child, was presented to us 10 h after a total amputation of the glans penis during a circumcision performed during the night in a pharmacy. Physical examination revealed a complete section of the glans and prepuce at the balanopreputial sulcus **(**Fig. 1**).** The section slice showed bleeding, and the child was hemodynamically stable. The investigation conducted to determine the circumstances of this accident revealed that the operator was a pharmacist. He was able to admit to us that he was alone without assistance to perform the procedure, and that the child was agitated and struggling, as if to justify himself. Having received the patient late and given the inadequate conditions of conservation of the glans, we decided to perform a meatoplasty. Admitted to the operating room and under general anesthesia, a meatoplasty was performed in three planes via 4/0 Vicryl sutures in separate stitches: spongiourethral suture on a charriere 10 urethral catheter, suture of the albuginea of the corpora cavernosa; suture of the skin and subcutaneous tissues was done via Vicryl sutures 4/0. The final appearance is shown in Fig. 2. The patient received antibiotic therapy for 10 days. The removal of the catheter was done at 21 days post-operation and the postoperative course were uneventful. After a 2-month follow-up, on physical examination, the patient was a well-looking boy with normal growth for his age. The glans penis and the coronal sulcus were absent. He had a pinhole urethral meatus with scaring at the distal end **(**Fig. 3**)** but adequate penile stump length. His parents reported normal voiding stream and examination revealed no stenosis. He had a good urine flow. Ultrasound showed normal urinary residue. The bladder wall was not thickened and there was no ureterohydronephrosis on ultrasound scan. His renal function was normal and urine culture did not isolate any organism.

## 3 Discussion

Circumcision is a surgical act and as such it must be practiced under safe conditions by trained hands. In many communities, particularly in sub-Saharan Africa where circumcision is commonly practiced, it still has a status of minor surgery**^2^****.** This allows paramedics as well as non-healthcare professionals to practice it under non-compliant conditions in return for the payment of “fees”. Being by far the most common surgical procedure most practiced in the world**^2^****,** for religious, ritual, socio-cultural, aesthetic and medical reasons, circumcision can be a source of numerous complications. The amputation of the glans that our report is one of the most tragic of all the complications of circumcision. Amputation of the glans after circumcision is a technical fault that should not happen. It can only be attributed to the operator as in our case.

Among other factors, the management of penile glans amputation depends on the duration before presentation and the acute phase management usually involves autotransplantation**^3^****.** In the first 8 h following circumcision, autotransplantation of the properly preserved glandular tissue is possible with significantly high success rate**^1^****.** It allows the restoration of the functional and aesthetic aspects of the glans. If management is delayed, or if the sectioned area is of poor quality, we simply close the corpora cavernosa and perform a meatoplasty in order to allow the patient to urinate in the best possible conditions. However, successful re-implantations have been reported 18 hours after amputation of the glans**^4^****.**

Unfortunately, most of the glans penis amputations seen in our setting are reported very late, well beyond 8 h, making any hope of successful glans re-implantation slim. This is due to the conditions of delivery of the glans penis and the difficulties of vascular anastomosis by microsurgery which is lacking in our structure. A meatoplasty was performed in our patient to improve urinary comfort and prevent infectious and mechanical complications of the urinary tract. The urethral meatus stenosis is the most common postoperative complication and may require dilatation or revision. Based on the clinical presentation, the goals of the management were to achieve a largely patent urethral opening in the long term and to prevent further urethral meatus stenosis.

The amputations of the penile glans lead to disturbances of a psychological nature**^5^****.** These can be related to the modification of the body schema and a loss of self-esteem. These serious repercussions can motivate subsequent repair attempts with the aim of making a neo-glans. We also suggest that the patient be referred to a psychologist for further consultation and support. The sexual function remains to be evaluated later in our patient who is still young.

## 4 Conclusion

The amputation of the glans is a serious complication of circumcision because it can engage the urinary and sexual prognosis. Early management is a reason to hope for a successful re-implantation of the glans. The amputation of the glans can be prevented by educating circumcisers.

## Consent

Written informed consent was obtained from the parents for publication.

## Authors' contributions

OS, CZO and A S: Drafting manuscript and bibliographic research through a review of the literature. B S, CBG and AKN: Correction and elaboration of the final manuscript. They constituted the ethics committee that approved the document. All the authors have read and agreed to the final version of the manuscript.

## Funding sources

This research is supported by research4Life with reference 3297.

## Declaration of competing interest

The authors have no conflicts of interest to declare.
